# The *Caenorhabditis elegans* *tmc-1* is involved in egg-laying inhibition in response to harsh touch

**DOI:** 10.17912/micropub.biology.000439

**Published:** 2021-08-16

**Authors:** Eva Kaulich, Denise S Walker, Yi-Quan Tang, William Ronald Schafer

**Affiliations:** 1 MRC Laboratory of Molecular Biology, Francis Crick Avenue, Cambridge CB2 0QH, UK

## Abstract

The conserved family of Transmembrane channel-like (TMC) proteins has attracted significant interest since two members appear to be key components of the mammalian hair cell mechanotransducer involved in hearing. *C. elegans* expresses two TMC proteins, TMC-1 and TMC-2. TMC-1 is widely expressed in in both muscles and the nervous system. This wide expression pattern suggests that TMC-1 might serve different functions in the various neurons. TMC-1 has previously been shown to function in neurons, playing a role in chemosensation in the ASH neurons and mechanosensation in OLQ neurons, further supporting this hypothesis. *tmc-1* is expressed in the high-threshold mechanosensory neuron, ALA. We show that *tmc-1* mutants show defects in the ALA-dependent inhibition of egg-laying in response to a harsh mechanical stimulus.

**Figure 1. TMC-1 is expressed in ALA, functions in ALA-dependent inhibition of egg-laying, but does not appear to function in response to harsh touch in ALA:**
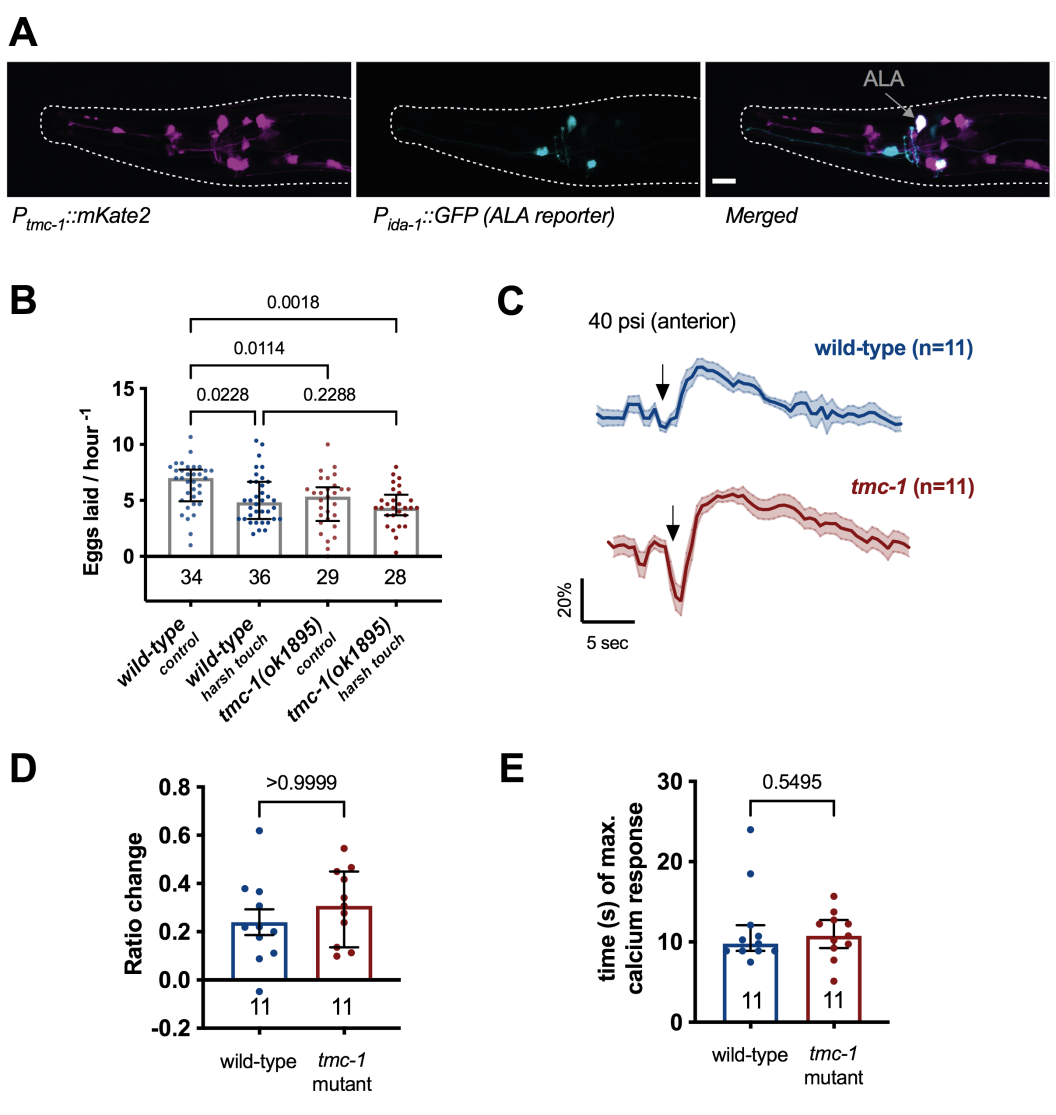
(A) A transcriptional reporter of *tmc-1* (*ljEx1221[Ptmc-1(4kb)::mKate2]*) is expressed in the ALA neuron (marked by *ljEx463 [Pida- 1::GFP]*). Scale bar 10μm. (B) Egg-laying rate in response to an anterior harsh touch. Each data point presents one individual animal, eggs were counted after 3 hours and normalised to one hour. A One-way ANOVA was statistically significant (p=0.0023), a post-hoc test (Bonferroni) revealed a significant difference between the response to anterior touch in *tmc-1* mutants and wild-type. (C) Wild-type (blue) and *tmc-1* mutant (brown) ALA calcium transients in response to a 1 second anterior harsh touch stimulus (arrow; 40psi). Mean and SEM are shown. (D) Maximum ratio change of GCaMP3 fluorescence shows no statistically significant difference between *tmc-1* mutants and the wild-type. A Mann-Whitney U test was non-significant. (E) Time to maximum after delivery of stimulus. Time measured from stimulus to the maximum of GCaMP fluorescence does not show statistically significant difference between *tmc-1* mutants and the wild-type. A Mann-Whitney U test was non-significant. Error bars for each panel represent median ± interquartile range (IQR) unless otherwise stated. Every data point presents an individual animal. The numbers above the graphs present *p*-values for the respective statistical test and number underneath the graph indicates number of total animals used for each condition.

## Description

The TMC proteins are a large family of integral transmembrane proteins which are conserved across the animal kingdom and have emerged as promising candidates for mechanotransducers (Keresztes *et al.*, 2003; Kurima *et al.*, 2002; Kurima *et al.*, 2003). They include eight proteins in mammals (Tmc1-8), one in *Drosophila* (TMC) and two in *C. elegans* (TMC-1; TMC-2) (Guo *et al.*, 2016; Keresztes *et al.*, 2003; Kurima *et al.*, 2002; Kurima *et al.*, 2003). Murine studies and human genetic screens have implicated TMCs in mechanotransduction, in particular *Tmc1*, which is an important deafness gene (see review: (Kawashima *et al.*, 2015)). Reintroducing the *Tmc2* gene can rescue sensory transduction and auditory function in mice carrying recessive *Tmc1* mutations, suggesting that they can perform redundant functions in inner cochlear hair cells, where they localise to the tips of stereocilia the site of hair cell sensory transduction (Askew *et al.*, 2015). In both *C. elegans* and *Drosophila melanogaster* TMCs show a broad expression pattern that is not restricted to sensory neurons (Guo *et al.*, 2016; Tang *et al.*, 2020; Yue *et al.*, 2018; Zhang *et al.*, 2015). *Drosophila* *tmc* has been shown to regulate larval locomotion, and expression of mammalian *Tmc1* or *Tmc2* in the *tmc*-expressing *Drosophila* neurons rescues mutant phenotypes (Guo *et al.*, 2016). *C. elegans* has two TMC genes, *tmc-1* and *tmc-2, that* are widely expressed in muscle and in the nervous system (Tang *et al.*, 2020; Yue *et al.*, 2018; Zhang *et al.*, 2015). Its wide expression pattern suggests that *tmc-1* might serve different functions in the various neurons. Until recently, *tmc-1*‘s only reported function in *C. elegans* neurons was in chemosensation (Chatzigeorgiou *et al.*, 2013; Wang *et al.*, 2016). However, we and others have identified *tmc-1* expression in *C. elegans* neurons involved in mechanotransduction, such as the OLQ neurons and the high-threshold mechanosensory neuron ALA (Tang *et al.*, 2020; Zhang *et al.*, 2015). The expression of *tmc-1* in ALA gives us the opportunity to investigate the conserved mechanosensory function of TMCs *in vivo*. *tmc-1* mutants accumulate more eggs and TMC-1 is known to function as a sodium leak channel in both the vulval muscles and the HSN egg laying command neurons, modulating membrane excitability (Yue *et al.*, 2018). However, ALA is required for the inhibition of egg-laying in response to a strong mechanical stimulus and mutations that impair ALA function abolish this harsh touch-induced inhibition (Sanders *et al.*, 2013). We therefore investigated whether TMC-1 functions in mechanotransduction in ALA.

***tmc-1* is expressed in the high-threshold mechanosensor ALA.** Previous research has identified*C. elegans* *tmc-1* gene to be expressed in different tissues such as the body wall muscles and neuronal tissue including sensory neurons, cholinergic ventral cord neurons, and pharyngeal neurons (Chatzigeorgiou *et al.*, 2013; Kratsios *et al.*, 2011; Tang *et al.*, 2020; Zhang *et al.*, 2015). To further investigate the role of TMC-1 in mechanosensory neurons, we firstly generated a new transcriptional reporter line for the endogenous *C. elegans* *tmc-1* promoter (4kb upstream of the start of the *tmc-1* gene) driving the red fluorescent protein *mKate2* (*ljEx1221 [Ptmc-1(4kb)::mKate2]*).We confirmed previous results that *tmc-1* is expressed in ALA (Zhang *et al.*, 2015) using a reporter strain carrying an ALA neuron marker (*ljEx463 [Pida-1::GFP])* (Fig. 1A).

***tmc-1* mutants have a defect in the inhibition of egg-laying in response to an anterior harsh touch stimulus**. Previous research has shown that ALA is a high-threshold mechanosensor which responds to harsh touch and is required for the inhibition of egg-laying in response to a harsh mechanical stimulus (Sanders *et al.*, 2013). Therefore, we investigated egg-laying behaviour of *tmc-1* mutants when undisturbed and when an anterior harsh touch was applied to the animal. In this assay, one animal is placed on a 30mm NGM plate, seeded with 10 μl *E. coli* OP50 at its centre. After acclimation, the experimental group received a manual anteriorly delivered harsh-touch stimulus using a metal worm pick every 20 minutes for 3 hours, while the control group remains undisturbed. After the three hours, the number of eggs on each plate is counted. Our results indicated that even under undisturbed control conditions *tmc-1* mutants laid significantly less eggs compared to the wild-type (Fig.1B). This is in line with previous research and consistent with its role in HSN and the vulva muscles (Yue *et al.*, 2018). However, while the wild-type showed a reduction of eggs laid after anterior harsh touch the *tmc-1* mutants’ egg-laying behaviour was unaffected, suggesting that TMC-1 does indeed function in the inhibition of egg-laying in response to harsh touch.

***tmc-1* mutants exhibit normal ALA calcium responses to anterior harsh touch.** Previous research showed that ALA responses to anterior stimulation resulted in a rapid increase to peak GCaMP3 fluorescence (Sanders *et al.*, 2013). These experiments used freely behaving animals, whereas we used a microfluidic chip which allowed precise and consistent stimulation but constrained the animals’ movement (Cho *et al.*, 2017). By comparing the calcium transients in the *tmc-1* mutants and wild-type animals in the ALA neurons, we found that both strains responded to harsh touch stimulation (40 psi) in the microfluidic device (Fig. 1 C). The responses to anterior harsh touch were different than previously reported (Sanders *et al.*, 2013) where responses were slower time to maximum, had a higher amplitude and longer duration compared to the responses that we observed. The differences can be explained by the different conditions and stimulus. We observed no difference in the response magnitude and no difference in the magnitude or time to maximum between the wild-type and the *tmc-1* mutant (Fig. 1 D, E).

Here we confirm that *tmc-1* is expressed in the harsh touch mechanosensory ALA neuron (Zhang *et al.*, 2015). In the wild-type a harsh mechanical stimulus using a worm-pick leads to the inhibition of egg-laying, which depends on the mechanosensory neuron ALA (Sanders *et al.*, 2013). While we saw a reduction in egg-laying in wild-type animals in response to anterior harsh touch, the *tmc-1* mutants did not show any inhibition but rather maintain the same egg-laying. This lack of inhibition is similar to what has been reported for the ALA defective *ceh-17* mutants, in which ALA function is impaired due to defects in axon-guidance (Sanders *et al.*, 2013). However, compared to the *tmc-1* mutants the egg-laying rate of *ceh-17* mutants is normal (Sanders *et al.*, 2013). Using mechanical stimulation in a microfluidic chip, we found that in *tmc-1* mutants ALA calcium transients were similar to the wild-type. These findings suggest that TMC-1 is not directly involved in sensing harsh touch in ALA. Unstimulated *tmc-1* mutants already lay fewer eggs, which might make it hard to detect further inhibition. Cell-specific rescue of *tmc-1* only in the egg-laying circuit or cell specific rescue of *tmc-1* in ALA might shed light on the function of *tmc-1* in ALA-dependent inhibition of egg-laying.

## Methods

*Animals.* All nematodes were grown at 22°C on 60mm NGM (nematode growth medium) agar plates seeded with the *Escherichia coli (E. coli)* OP50 strain. The following strains were used:the wild-type (Bristol N2), AQ4537 *tmc-1(ok1859)* 6x times outcrossed*,* AQ4330 (*ljEx1221[Ptmc-1(4kb)::mKate2]*) crossed with AQ2908 (*ljEx463 [Pida-1::gfp*]) for confirmation of *tmc-1* expression in ALA, INV21001 (*Ex[Pver-3::GCaMP3])* for the calcium imaging experiments.

*Molecular biology.* Plasmid pEK158 (*Ptmc-1(4kb)::mKate2*) was generated using genomic DNA, amplified from lysed worms, and assembled using Gateway Cloning® (Thermo Fisher Scientific), using SnapGene for primer design..

*Egg-Laying Assay*. Day 1 adults were used in the assay previously described (Sanders *et al.*, 2013), at room temperature. Briefly, plates with the different strains were blinded to avoid bias.15 animals were used for each condition in each assay and the assay was repeated three times, however, some animals crawled under the agar or off the plate, and were therefore disqualified. Using an eye-lash, a single animal was placed on a 30mm NGM plate, seeded with 10 μl *E. coli* OP50 at its centre 1.5 hours prior to start of the assay. The assay time was 3 hours, during which animals in the experimental group received a manual anteriorly delivered harsh touch stimulus using a metal worm pick every 20 minutes. The control group was not stimulated. After the three hours, the adults were removed and the number of eggs on each plate was counted.

*Microfluidic Calcium Imaging.* ALA calcium transients were assessed using the genetically encoded calcium indicator (GCaMP3), expressed under an ALA promoter in a microfluidic device adapted to test mechanosensory stimuli (Cho *et al.*, 2017). Day 2 adults were imaged using a previously established protocol by (Cho *et al.*, 2017). Briefly, using a hair-pick, animals were gently picked off food and transferred in a 1.5ml Eppendorf tube filled with S-Basal, then transferred to the microfluidic chamber. Before imaging, the animals loaded into the chip were left to acclimatise for 2 minutes before 40 psi mechanical stimulation was applied. The following stimulus protocol was used in the “Mechano-Chip” (Cho *et al.*, 2017): Total program time was 30 seconds, the mechanical stimulus of 40 psi (harsh touch stimulating) was delivered for the duration of one second at 10 seconds after onset of recording. Recordings were approximately three frames per second (fps). Experiments were performed on a Leica Axiovert 135 inverted microscope using a 40x air objective (N.A. 0.75). Video sequences were captured using a Hamamatsu ORCA-R2 (C10600-10B) camera with 100 ms exposure time. Simultaneous dual colour imaging was performed using an OptoSplit II (Andor Technology) beamsplitter containing a GFP(520 nm). CoolLED’s pE-300white was used as a light source for fluorescent imaging. Imaging was carried out in S-basal buffer (100mM NaCl, 0.05M phosphate buffer pH 6.0, 5 μg/mL cholesterol).

For analysis of calcium transients, fluorescence intensities for each frame were extracted using a custom MATLAB script (Cho *et al.*, 2018; Cho *et al.*, 2017). GCaMP3 intensities were measured as the mean pixel intensity of the 100 brightest pixels in a circular region of interest (ROI) with a 14-pixel radius. Calcium traces were computed as the change in R from the baseline value which were computed as the mean R prior to stimulus onset (Chew *et al.*, 2018; Cho *et al.*, 2018; Cho *et al.*, 2017).The ALA maximum response was calculated in Excel by subtracting the baseline (average of fluorescence for the first 5 seconds of recording) from the maximum value (determined as the maximum value after the stimulus). These values were plotted in GraphPad Prism.

*Statistical Analysis.* Statistical Analysis was carried out in GraphPad Prism version 9.0.2 for macOS, GraphPadSoftware, San Diego, California USA (www.graphpad.com). Normality was assessed by a Shapiro-Wilk test. If thedata followed a normal distribution, a parametric test was deployed, if not, the nonparametricequivalent was chosen. Appropriate post-hoc tests were always used formultiple comparisons (Bonferroni correction). The parameters and statistical test usedare indicated in each figure description.
